# Case Report: Ultrasound Guided Mucosal Fold Lateralization for Laryngeal Webs

**DOI:** 10.3389/fsurg.2021.781422

**Published:** 2021-11-19

**Authors:** Sohit Paul Kanotra

**Affiliations:** ^1^Department of Otolaryngology—Head and Neck Surgery, University of Iowa Hospitals and Clinics, Iowa, IA, United States; ^2^Stead Family Children's Hospital, Iowa, IA, United States

**Keywords:** pediatric, laryngeal web, tracheostomy, ultrasound, airway, stridor, laser, suture laterization

## Abstract

The surgical management of Laryngeal webs is challenging and is associated with a high recurrence rate due the presence of opposing raw mucosal surfaces of the vocal cords, especially near the anterior commissure which causes re-scarring. We describe an endoscopic technique of mucosal flap lateralization (MFL) with ultrasound guidance, which prevents the apposition of the anterior raw surfaces of the vocal cords after web incision, thus avoiding recurrence.

## Introduction

Congenital Laryngeal webs (LWs) are rare entities and are classified based on the percentage of vocal cord involvement and the presence of subglottic extension as given by Cohen (1–3). Presentation is dependent on the type of LW and can range from an incidental finding to mild hoarseness, weak cry, stridor, dyspnea, aphonia and severe respiratory distress requiring tracheostomy (1, 2). A multitude of endoscopic and open airway techniques have been described for the management of LWs including endolaryngeal web incision with placement of a keel or plate, laryngeal mucosal flap or grafts and open airway reconstructive techniques including laryngofissure with placement of a keel and laryngotracheal reconstruction with grafting and stent placement (4). The surgical management is challenging and is associated with a high recurrence rate reported to be 76% for endoscopic and 89% for open procedures (2). A major factor contributing to this is the presence of opposing raw mucosal surfaces of the vocal cords, especially near the anterior commissure which causes re-scarring.

We describe an endoscopic technique of mucosal flap lateralization (MFL) with ultrasound guidance, which prevents the apposition of the anterior raw surfaces of the vocal cords after web incision, thus avoiding recurrence.

## Surgical Technique

**Figure 1:**
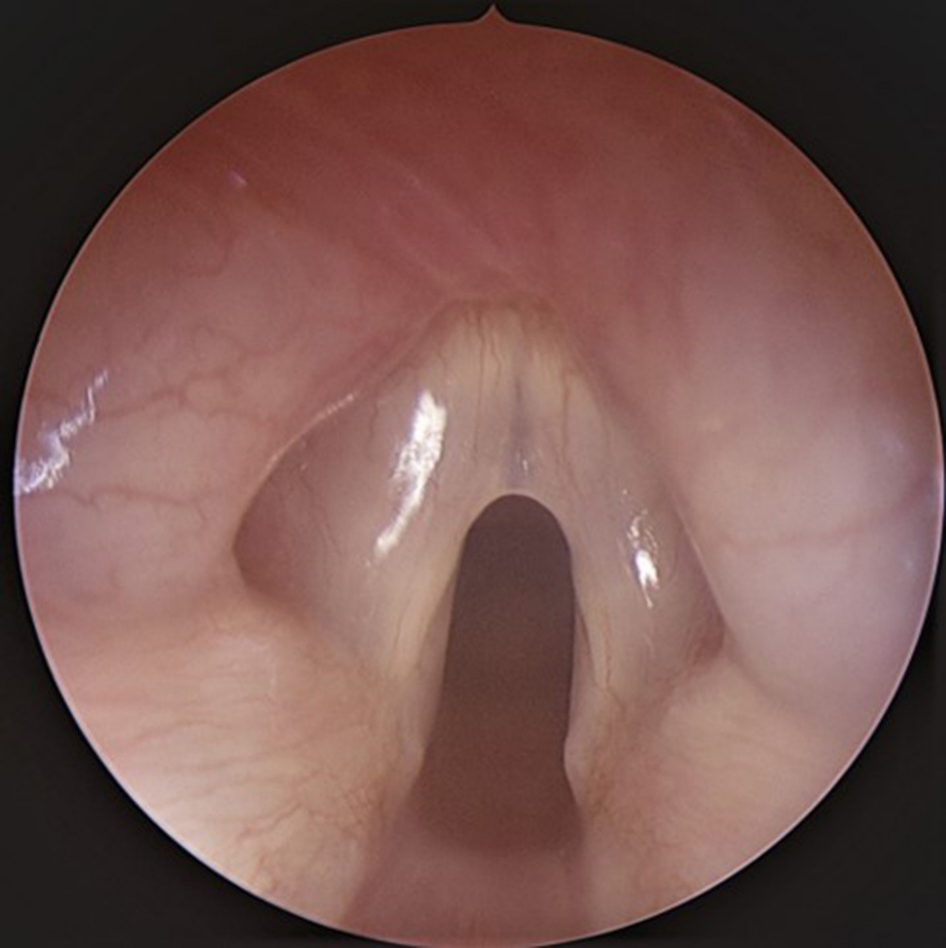
Endoscopic view of Type 2 laryngeal web.

The present surgical technique is described in a 15-month-old otherwise healthy child with Type 2 Laryngeal web (Figure 1) who presented with a weak voice. The case was retrospectively reviewed under University of Iowa IRB No: 20210205.

The surgical technique can be viewed in the Supplementary Video.

**Figure 2:**
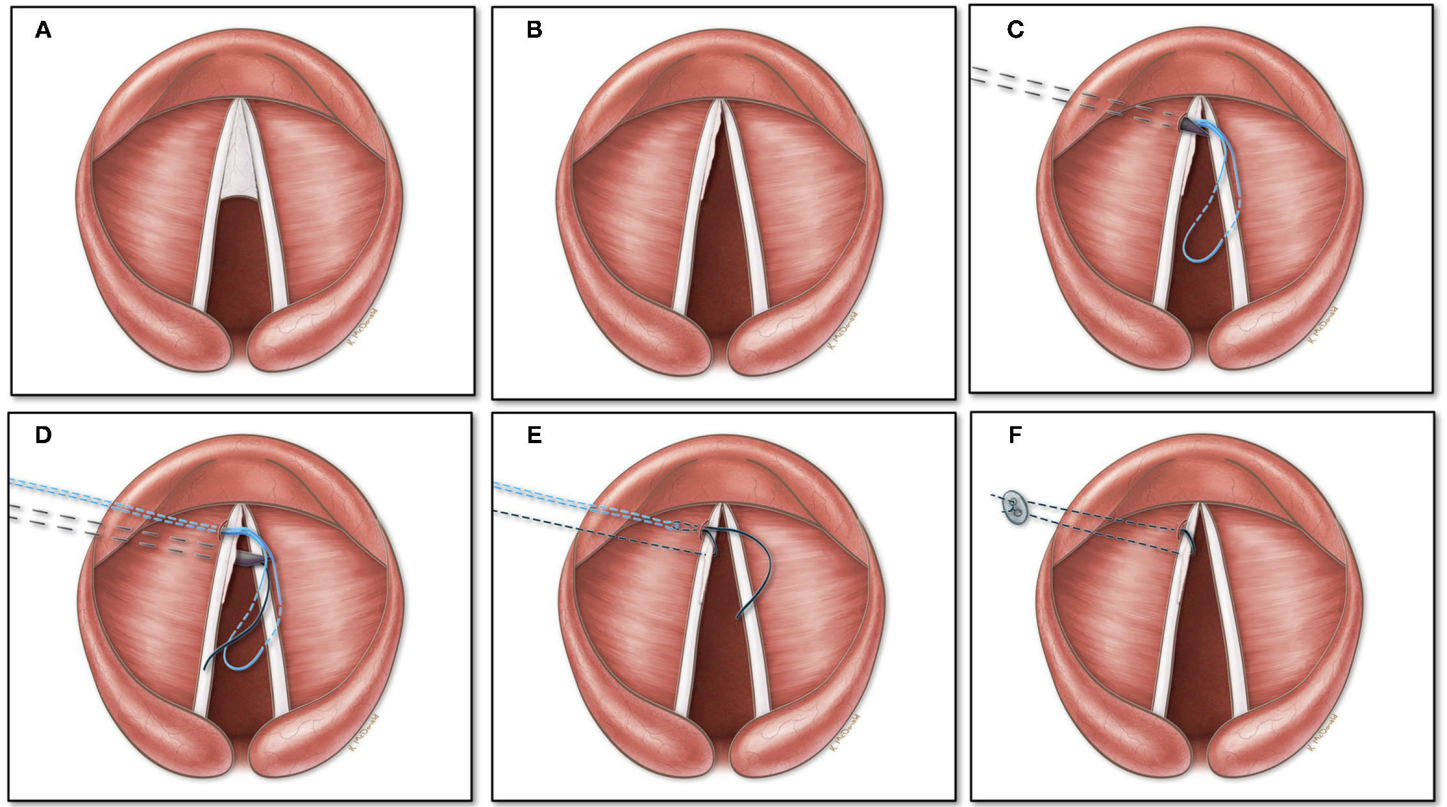
Mucosal flap laterization for laryngeal web: **(A)** Schematic view of Type 2 laryngeal web. **(B)** Remnant mucosal flap after unilateral laser incision of the laryngeal web. **(C)** An 18-gauge needle with a pre threaded looped 3–0 prolene suture is passed under ultrasonographic guidance targeting superior to the true vocal fold near the anterior commissure and is pulled out endolaryngeally. **(D)** A 20-gauge needle with a 3–0 prolene suture is placed immediately inferior to the true vocal cord under ultrasound guidance and threaded into the loop. **(E,F)** The looped stitch is pulled out through the neck, which carries the single stitch out, placing the suture with the mucosal flap wrapped on the left vocal cord.

The surgery is performed with the patient under spontaneous ventilation using a combination of propofol and sevoflurane. After direct laryngoscopy and rigid bronchoscopy, an appropriately sized Benjamin Lindholm laryngoscope (Karl Stroz, Germany) is introduced for laryngeal exposure which is placed in suspension with the aid of a self-retaining laryngoscope holder (Karl Storz Endoscopy-America, Inc.) secured to a Mayo stand. A vocal fold spreader is placed in order to completely expose the anterior glottic web till the anterior commissure. The microscope is then brought into the field and after implementation of all laser safety precautions, a straight handheld CO2 laser set at 2W, 20mj ultrapulse repeat mode (Lumenis, Inc) is used to incise the laryngeal web close to the edge of the right vocal fold (Figures 2A,B).

**Figure 3:**
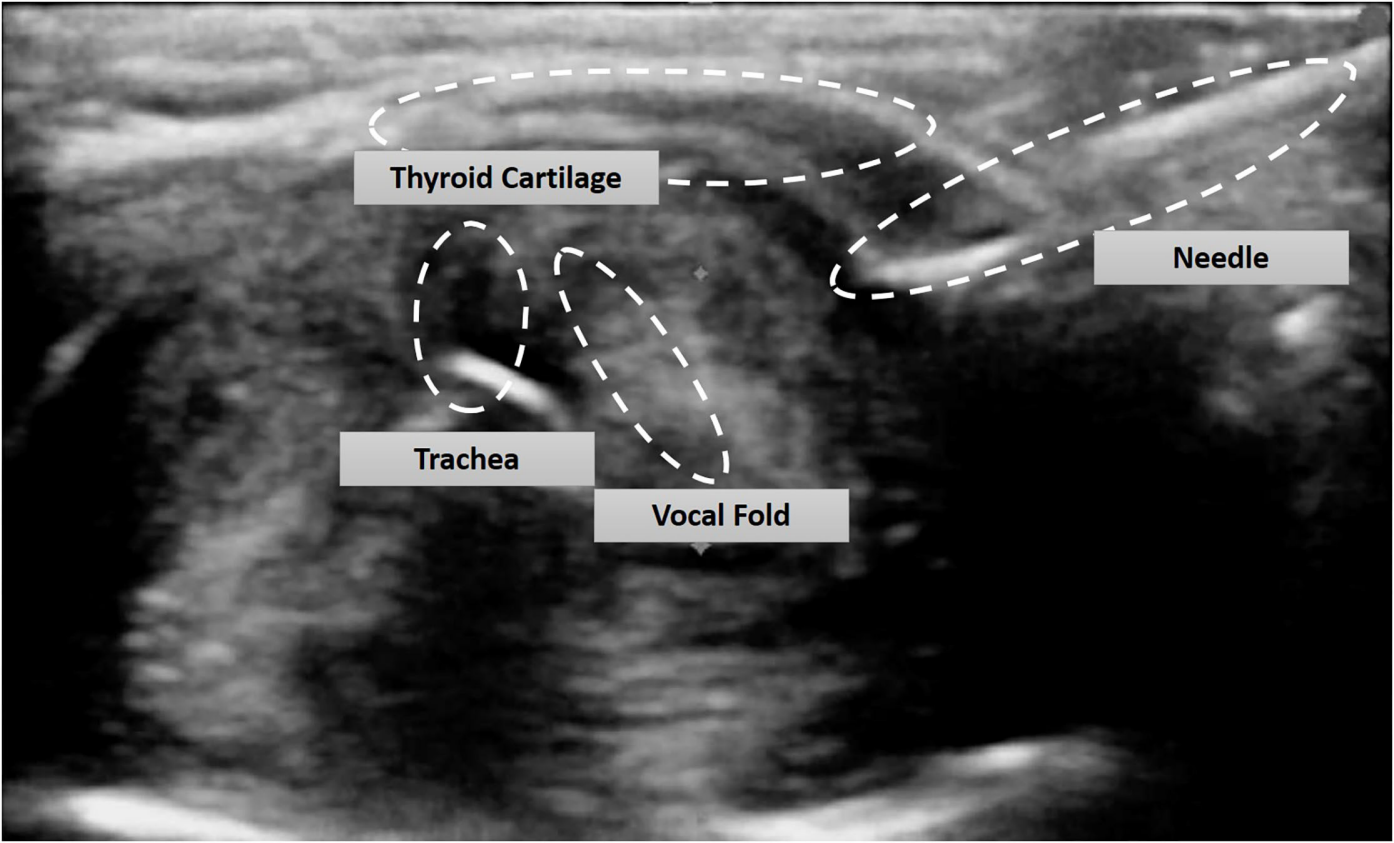
Axial ultrasound of the larynx with in-axis placement of the 18 G needle.

**Figure 4:**
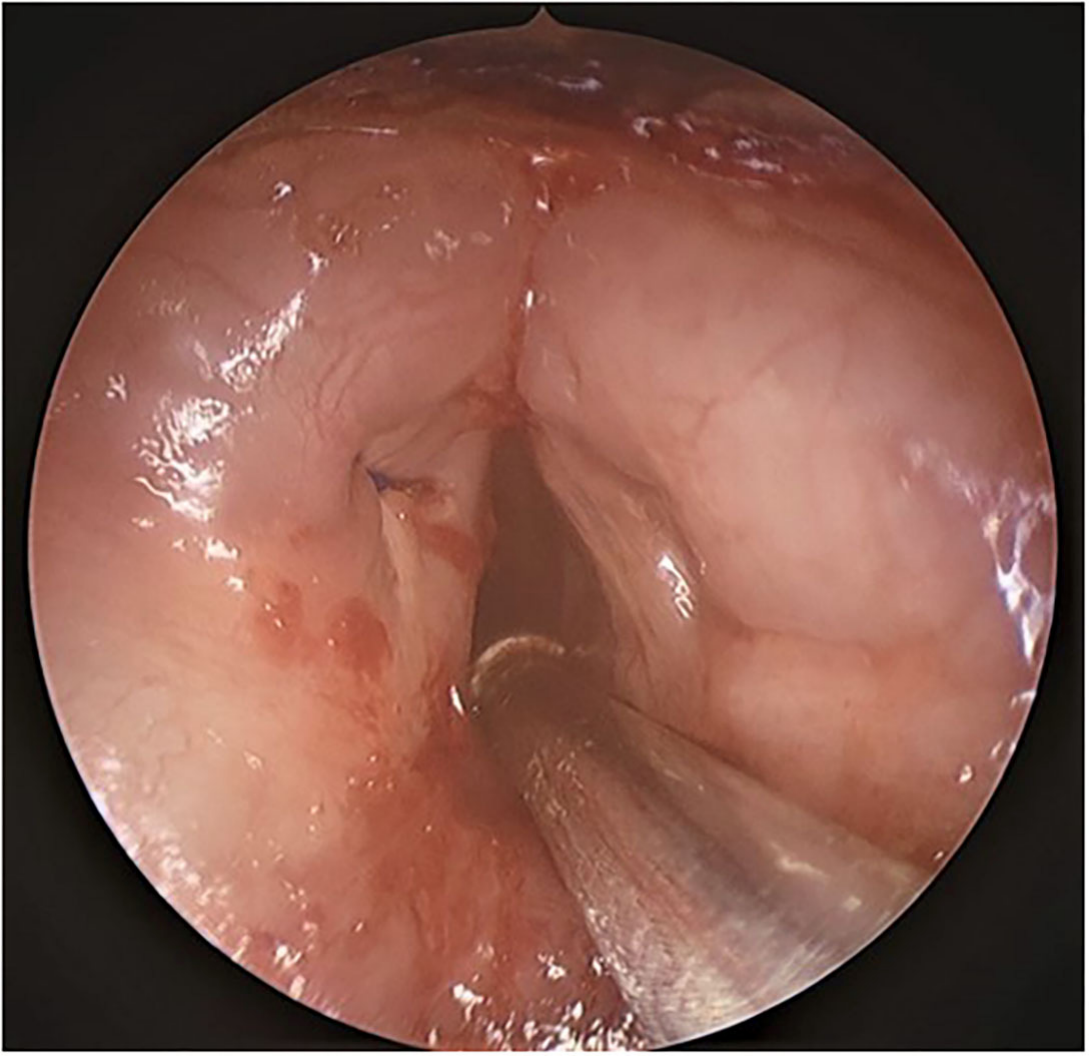
Endoscopic view of the mucosal flap laterization. Suture is visible on the left vocal cord.

The thin mucosal flap is then subsequently wrapped on to the left vocal fold which is then lateralized using an ultrasound guided exo-endolaryngeal technique of suture lateralization, which we have described in detail previously (5). In brief after prepping and draping the neck, an ultrasound machine (FUJIFILM Sonosite, Bothell, Ishington, USA) is brought into the field and a sterile sleeve is applied to the L25 probe. After identification of landmarks, the ultrasound probe is positioned to provide an axial plane view of the neck and larynx. A 5 mm neck incision at the level of the true vocal fold, 1.5 cm lateral to the midline is performed on the left with creation of subcutaneous pocket. An 18-gauge needle with a pre threaded looped 3–0 prolene suture (polypropylene; Ethicon, Somerville, NJ) is placed into the neck incision site and advanced along the axial plane under ultrasonographic guidance targeting superior to the true vocal fold anteriorly near the anterior commissure (Figures 2C, 3). The needle is identified endoluminally, and the suture is pushed through the needle which is drawn superiorly with a grasper until taken out through the laryngoscope (Figure 2D). The needle is removed. Next, a 20-gauge needle with a 3–0 prolene suture end threaded inside the bevel of the needle is placed immediately inferior to the true vocal cord under ultrasound guidance (Figure 2E). The suture is advanced, grasped and removed through the laryngoscope and threaded into the loop while holding tension on the single suture as the loop exits the neck. The looped stitch is pulled out through the neck, which carries the single stitch out placing the suture with the mucosal flap wrapped on to the left vocal cord (Figures 2F, 4). Two small puncture holes are then made in a trimmed 0.005 thickness silastic sheet which is placed within the wound pocket and the suture knot is tied onto the sheet. The suture is tied with sufficient tension to achieve minimal lateralization. The skin is reapproximated and the patient is then transferred to pediatric intensive care unit for observation. After 1 week, the suture along with the silastic sheet is removed under anesthesia and the remainder of the mucosal flap on the left vocal fold is excised using the same laser settings as previously used.

## Result

**Figure 5:**
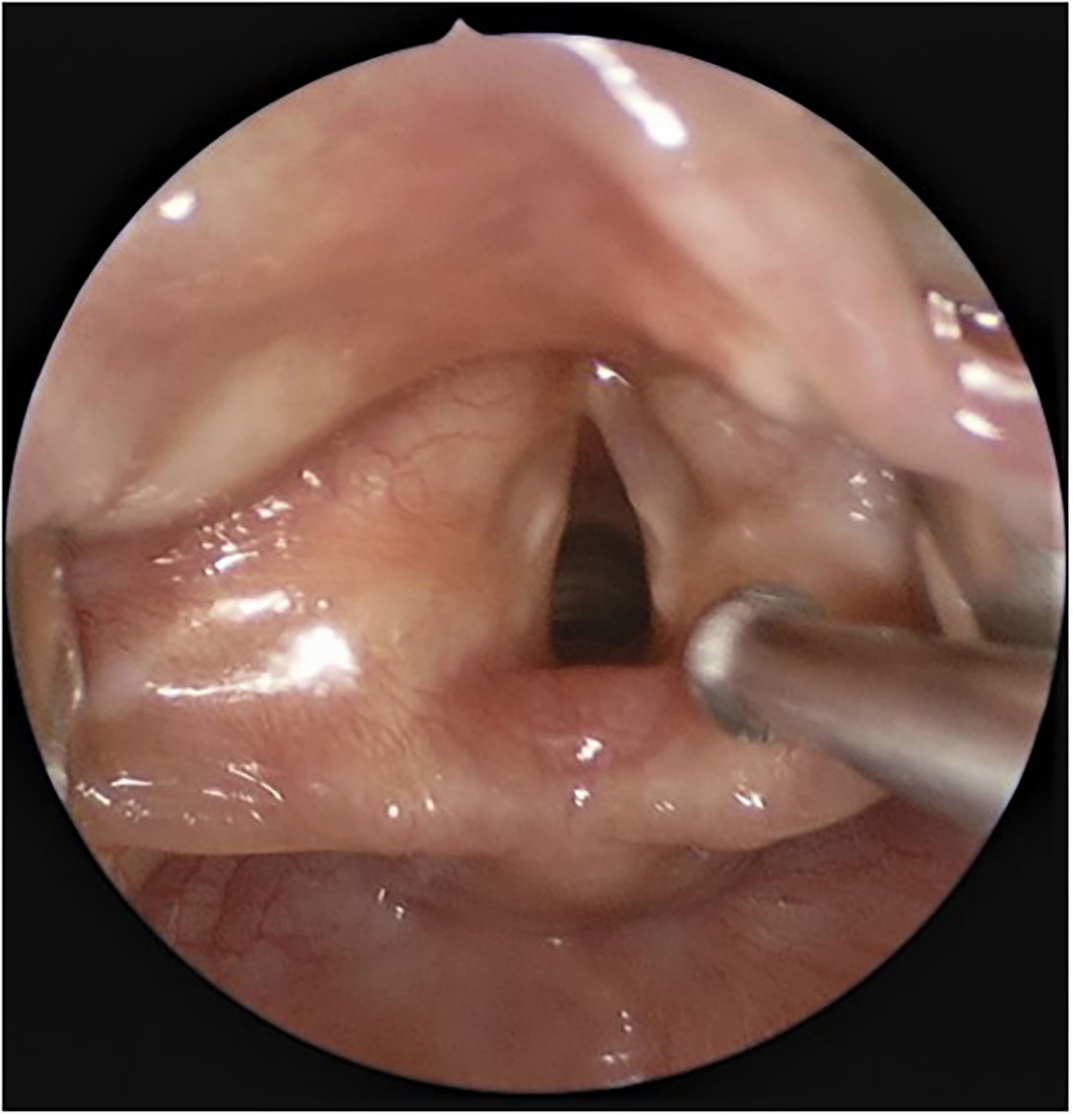
Endoscopic view at 2 month follow up with no evidence of recurrence of the Laryngeal web.

A 2 months follow up, revealed no evidence of recurrence of the laryngeal web with significant subjective improvement in voice (Figure 5). The result has been the same at a recent 6 month follow up.

## Discussion

The biggest challenge in the endoscopic management of LWs is to prevent apposition of the anterior vocal folds to avoid rewebbing. The present technique of mucosal flap lateralization provides a method of maintaining distance between the anterior vocal cords, preventing contact of raw mucosal surfaces. The use of ultrasound guidance for accurately predicting the trajectory of the needle assists in precise placement of the endolaryngeal stitch avoiding multiple passes and preventing injury to the delicate laryngeal mucosa.

A multitude of surgical techniques have been described for avoiding juxtaposition of the anterior vocal cords after LW excision. The most widely used is endoscopic excision and placement of a silicon keel. However, keel placement has been associated with a high recurrence rate of 10–30% (4). Also, keel placement is technically challenging endolaryngeally, in the narrow confines of the pediatric larynx and can be associated with formation of new granulation tissue, necrosis and scarring while having an inherent risk of acute airway obstruction in the absence of a tracheostomy (4). Endolaryngeal mucosal flaps or free graft to cover one or both raw surfaces of the anterior vocal cord have also been described, however endolaryngeal suturing of the mucosal flaps and free grafts is technically challenging and results in irreversible anatomical changes to the vocal cords (1, 6).

The present technique provides a way of keeping the anterior vocal fold raw mucosal surfaces separated till complete healing has taken place of the contralateral vocal cord. The ultrasound guided exo-endolaryngeal technique of suture lateralization has been previously described by our team for the management of bilateral vocal cord paralysis (5). We have also used the exo-endolaryngeal lasso technique for epiglottopexy for management of epiglottic prolapse (7). The technique is easy to perform and can be achieved by regular surgical needles without the need of specialized equipment. There are some surgical nuances which need to be emphasized to achieve optimal results.

The authors preference is to use flexible fiber CO2 laser for the incision of the LW, however cold steel microlaryngeal instrumentation can also be used to achieve similar result. The ultrapulse mode of laser emission provides control and precision while reducing the damage to the surrounding tissues. The flexible fiber laser helps in greater maneuverability in the limited space of the pediatric larynx. Preloading the needles with sutures and making sure that they can easily slide through the needles will ensure that the surgical procedure runs smoothly. The external puncture site is critical and should be 1.5 cm lateral to the midline at the level of the vocal cords which can be determined with the help of ultrasound guidance. The ultrasound probe is used in the axial plane with an in-line axis needle approach which allows for visualization of the whole trajectory of the needle, accurately placing the needle in the anterior part of the vocal cord, while preventing repeated passes. The suture is tightened over a silastic button to avoid cutting through the soft tissue and buried underneath the strap muscles to avoid extrusion and stitch abscess. While tightening the suture it is important that the stitch is being visualized endolaryngeally to achieve minimal laterization to avoid increased separation of the vocal cord which might lead to aspiration. For LWs with greater glottic involvement multiple sutures can be placed in order to achieve mucosal flap lateralization. The simplicity of the technique makes it an option for management of children with predominant voice symptoms rather than waiting in this subset of patients till school age.

The major drawback of the technique is the possibility of producing aspiration. In the present case transient choking was noticed which resolved after adequate pain management. Even if postoperative aspiration is noticed, it will be transitory, as the suture is cut within a week of placement. Another surgical risk is the possibility of glottic and subglottic trauma secondary to multiple needle passes. However, with the assistance of ultrasound, we were able to complete the procedure in a single needle pass. The technique is also a two-stage procedure and warrants a second stage to take down the suture and excise the redundant mucosa on the left vocal fold. However, most techniques described for the management of LWs require a second stage for removal of an endoscopically placed keel. Also, the technique is of limited value in case of subglottic extension and hence is not an option for Type 4 LWs.

Future studies using a larger sample size and outcome analysis regarding the tissue impact, and technical ease should be undertaken to demonstrate the effectiveness of this technique.

## Conclusion

We propose an ultrasound guided mucosal flap lateralization technique for management of laryngeal webs which is a simple way of avoiding vocal cord apposition and preventing recurrence of LWs.

## Data Availability Statement

The original contributions presented in the study are included in the article/Supplementary Material, further inquiries can be directed to the corresponding author/s.

## Ethics Statement

The studies involving human participants were reviewed and approved by University of Iowa IRB No: 20210205. Written informed consent to participate in this study was provided by the participants' legal guardian/next of kin.

## Author Contributions

SK contributed to the idea, writing of the manuscript, and final approval.

## Conflict of Interest

The author declares that the research was conducted in the absence of any commercial or financial relationships that could be construed as a potential conflict of interest.

## Publisher's Note

All claims expressed in this article are solely those of the authors and do not necessarily represent those of their affiliated organizations, or those of the publisher, the editors and the reviewers. Any product that may be evaluated in this article, or claim that may be made by its manufacturer, is not guaranteed or endorsed by the publisher.

## References

[1] Kuo IC Rutter M. Surgical management of anterior glottic webs. Front Pediatr. (2020) 8:555040. 10.3389/fped.2020.555040 33194889 PMC7604345

[2] Lawlor CM Dombrowski ND Nuss RC Rahbar R Choi SS. Laryngeal web in the pediatric population: evaluation and management. Otolaryngol Head Neck Surg. (2020) 162:234–40. 10.1177/0194599819893985 31842676

[3] Cohen SR. Congenital glottic webs in children. A retrospective review of 51 patients. Ann Otol Rhinol Layngol Suppl. (1985) 121:2–16. 10.1177/00034894850940S601 3935032

[4] Izadi F Delarestaghi MM Memari F Mohseni R Pousti B Mir P. The butterfly procedure: a new technique and review of the literature for treating anterior laryngeal webs. J Voice. (2010) 24:742–9. 10.1016/j.jvoice.2009.03.005 19850447

[5] Puricelli MD Peterson J Kanotra SP. Ultrasound-guided suture lateralization in pediatric bilateral vocal fold immobility. Laryngoscope. (2020) 130:E941–4. 10.1002/lary.28553 32083723

[6] Deganello A Gallo O Gitti G de'Campora E Mahieu H. New surgical technique for endoscopic management of anterior glottic web. Acta Oto-Rhino-Laryngologica Belgica. (2010) 6:261. 21302688

[7] Kanotra SP. Transcervical epiglottopexy for the management of type 3 laryngomalacia. Ear, Nose & Throat Journal. (2020) 4:0145561320971952. 10.1177/0145561320971952 33147060

